# Which measuring site in ankylosing spondylitis is best to detect bone loss and what predicts the decline: results from a 5-year prospective study

**DOI:** 10.1186/s13075-017-1480-0

**Published:** 2017-12-08

**Authors:** Anna Deminger, Eva Klingberg, Mattias Lorentzon, Mats Geijer, Jan Göthlin, Martin Hedberg, Eva Rehnberg, Hans Carlsten, Lennart T. Jacobsson, Helena Forsblad-d’Elia

**Affiliations:** 1 Department of Rheumatology and Inflammation Research, Sahlgrenska Academy at University of Gothenburg, Box 480, 405 30 Gothenburg, Sweden; 2 Geriatric Medicine, Institute of Medicine, Sahlgrenska Academy at University of Gothenburg and Sahlgrenska University Hospital, 413 45 Gothenburg, Sweden; 3 Department of Radiology, Faculty of Medicine and Health, Örebro University, 701 82 Örebro, Sweden; 4 Department of Clinical Sciences, Lund University, Box 117, 221 00 Lund, Sweden; 5 Department of Radiology, Sahlgrenska University Hospital/Mölndal, 431 80 Mölndal, Sweden; 6 Section of Rheumatology, Södra Älvsborg Hospital, 501 82 Borås, Sweden; 7 Section of Rheumatology, Alingsås Hospital, 441 33 Alingsås, Sweden; 8 Department of Public Health and Clinical Medicine, Rheumatology, Umeå University, 901 87 Umeå, Sweden

**Keywords:** Ankylosing spondylitis, Osteoporosis, Bone mineral density, Inflammation, Longitudinal study

## Abstract

**Background:**

Studies have shown increased prevalence of osteoporosis and increased risk for vertebral fractures in patients with ankylosing spondylitis (AS). Measurements of bone mineral density (BMD) in the lumbar spine anterior-posterior (AP) projection may be difficult to interpret due to the ligamentous calcifications, and the lateral projection might be a better measuring site. Our objectives were to investigate BMD changes after 5 years at different measuring sites in patients with AS and to evaluate disease-related variables and medications as predictors for BMD changes.

**Methods:**

In a longitudinal study, BMD in Swedish AS patients, 50 ± 13 years old, was measured with dual-energy x-ray absorptiometry (DXA) at the hip, the lumbar spine AP and lateral projections, and the total radius at baseline and after 5 years. Patients were assessed with questionnaires, blood samples, and spinal radiographs for grading of AS-related alterations in the spine with the modified Stoke Ankylosing Spondylitis Spinal Score (mSASSS) and assessment of vertebral fractures by the Genant score. Multiple linear regression analyses were used to investigate predictors for BMD changes.

**Results:**

Of 204 patients included at baseline, 168 (82%) were re-examined after 5 years (92 men and 76 women). BMD decreased significantly at the femoral neck and radius and increased significantly at the lumbar spine, both for AP and lateral projections. Mean C-reactive protein during follow-up predicted a decrease in the femoral neck BMD (change in %, β = –0.15, *p* = 0.046). Use of bisphosphonates predicted an increase in BMD at all measuring sites (*p* < 0.001 to 0.013), except for the total radius. Use of tumor necrosis factor inhibitors (TNFi) predicted an increase in AP spinal BMD (β = 3.15, *p* = 0.012).

**Conclusion:**

The current study (which has a long follow-up, many measuring sites, and is the first to longitudinally assess the lateral projection of the spine in AS patients) surprisingly showed that lateral projection spinal BMD increased. This study suggests that the best site to assess bone loss in AS patients is the femoral neck and that inflammation has an adverse effect, and the use of bisphosphonates and TNFi has a positive effect, on BMD in AS patients.

## Background

Ankylosing spondylitis (AS) is a chronic, inflammatory disease mainly affecting the sacroiliac joints, the axial skeleton, and sometimes the peripheral joints. Two bone-remodeling processes in AS include both new bone formation with development of syndesmophytes in the spine and bone loss with increased risk for osteoporosis and fractures. Studies have demonstrated increased prevalence of osteoporosis in patients with AS compared with sex- and age-matched controls [1, 2]. Low bone mineral density (BMD) of the lumbar spine and femoral neck has been observed in early AS and mild disease [2, 3]. AS patients also have an increased risk for vertebral fractures with a risk of instability and neurological injuries [4, 5].

Dual-energy x-ray absorptiometry (DXA) is the routine method for assessing BMD [6]; however, measurements at the anterior-posterior (AP) projection of the spine in AS patients may be difficult to interpret due to ligamentous calcifications superimposed on the vertebrae or to sclerosis of the vertebral endplates [1, 7]. The lateral projection of the spine supposedly more selectively measures the trabecular-rich vertebral body without contribution from cortical-rich posterior spinal elements [8]. There are some longitudinal studies on changes in BMD in AS [9–13]. To our knowledge, no reports on changes over time in the lateral projection of the spine have been published. In two studies on older men the lateral projection showed decreasing BMD over time whereas AP BMD increased [14, 15].

In some previous longitudinal studies on BMD in AS, a decrease in BMD was observed, especially in patients with active disease [9–11, 13]. However, in order to give the patients optimal care more information is needed regarding predictors for osteoporosis and which measuring sites are best for diagnosis and monitoring of osteoporosis in this patient group. The aim of this study was to investigate how BMD changed over 5 years at five different measuring sites including the lateral projection of the spine and to evaluate disease-related variables and pharmacological treatments as predictors for the changes in BMD.

## Methods

### Patients

The patients were recruited at baseline in 2009 from rheumatology clinics at Sahlgrenska University Hospital in Gothenburg, Södra Älvsborg Hospital in Borås, and Alingsås Hospital, Sweden. The inclusion criterion was AS according to the modified New York criteria [16]. Exclusion criteria were psoriasis, inflammatory bowel disease, dementia, pregnancy, and difficulties in understanding the Swedish language. The 204 patients that completed the baseline protocol were invited to participate in the 5-year follow-up. Written informed consent was obtained and the study was approved by the regional ethics committee.

### Physical examination and questionnaires

All physical examinations were repeated at follow-up, including the Bath Ankylosing Spondylitis Metrology Index (BASMI). Examinations were performed by one physician (EK) at baseline and one physician (AD) at follow-up. Patients answered a questionnaire concerning risk factors for osteoporosis, medical history, and medication. The Bath Ankylosing Spondylitis Disease Activity Index (BASDAI), the Bath Ankylosing Spondylitis Functional Index (BASFI), and the Bath Ankylosing Spondylitis Patient Global score (BAS-G) were obtained [17–20]. The Ankylosing Spondylitis Disease Activity Score based on C-reactive protein (ASDAS-CRP) was calculated [21, 22]. The amount of tobacco smoking for current and ever smokers was estimated by smoking pack years, calculated by multiplying the number of packs of cigarettes smoked per day by the number of years the person had ever smoked. Data on nonsteroidal anti-inflammatory drug (NSAID) consumption during follow-up was collected according to the Assessment of SpondyloArthritis International Society (ASAS) recommendations [23]. Use of glucocorticoids, converted into milligrams of prednisolone, and duration of treatment with tumor necrosis factor inhibitors (TNFi) and bisphosphonates were estimated from the medical records. Use of TNFi or bisphosphonate was calculated by dividing the number of months of exposure to either medication with follow-up time in months for all patients, resulting in a value 0–1.

### Bone mineral density

BMD measurements from the lumbar spine in the AP (vertebrae L1–L4) and lateral (L2–L4) projections, the left hip (femoral neck and total hip), and the non-dominant forearm (total radius) were obtained using the same DXA scanner (Hologic Discovery A, Hologic Inc., Bedford, MA, USA) at baseline and at follow-up. Precision as a percentage coefficient of variation (CV) for repeated DXA measurements was 0.8% in the femoral neck, 0.6% in the total hip, 0.3% in the AP lumbar spine, 1.3% in the lateral lumbar spine, and 3.1% in the total radius. The World Health Organization (WHO) definitions for osteoporosis and osteopenia were used for patients ≥ 50 years; osteoporosis, T-score ≤ –2.5 SD (compared to the young normal mean) and osteopenia, T-score < –1 to > –2.5 SD [6]. For patients < 50 years a Z-score ≤ –2.0 SD (compared to age- and sex-matched mean) was considered to be below the expected range for age [24] and a Z-score < –1 SD to be subnormal. T- and Z-score reference values were provided by the DXA scanner manufacturer. The Hologic reference database consists of more than 45,000 observations from the USA and the current study used reference material from non-Hispanic white adults from the USA. Volumetric BMD (vBMD), a three-dimensional mode to assess bone mineral content per volume, was estimated by combining AP with lateral DXA scanning of the lumbar spine. Reference values were not available for lateral lumbar spine DXA for men or for vBMD for either sex.

### Radiography

Lateral radiographs of the spine were acquired at baseline and at follow-up. Osteoproliferative changes related to AS were assessed in the cervical and lumbar spine by the modified Stoke Ankylosing Spondylitis Spine Score (mSASSS). The score ranges from 0 to 72 [25]. The radiographs were also assessed for vertebral fractures (VFs) by the semiquantitative method Genant score. Vertebrae T4–L4 were assessed for reductions in height and graded 0 = normal, 1 = mild, 2 = moderate, and 3 = severe VF based on how large was the height reduction [26]. A progress in Genant score was defined as development of a new fracture in a previously normal vertebra or a worsening of at least 1 point in Genant score. All radiographs at baseline and follow-up were assessed by the same musculoskeletal radiologist (MG).

### Laboratory tests

Blood samples were analyzed using standard laboratory techniques. The mean level of erythrocyte sedimentation rate (ESR) and C-reactive protein (CRP) for the last 5 years before follow-up were obtained from the medical records. Mean ESR/CRP was calculated using the first recorded test for each year unless the patient had an infection; in that case the ESR/CRP was replaced by the subsequent test.

### Statistics

Statistical analyses were performed using IBM SPSS Statistics 22 (IBM Corp., Armonk, NY, USA). Descriptive statistics are presented as mean and standard deviation (SD) or standard error of the mean (SEM). To compare different groups the *t* test or the Mann-Whitney *U* test were used for continuous variables, and the Chi-square test used for categorical variables. For repeated measurements, a paired *t* test or the Wilcoxon rank sign test were used for continuous variables, and McNemar’s test for categorical variables. A one-sided *t* test was used to compare the Z-score in patients to the test value 0. The Δ values were calculated by subtracting the baseline value from the follow-up value. Standard multiple linear regression analyses were run with ΔBMD at the different measuring sites as a dependent variable. Predictor variables used in the models were demographic variables known to affect BMD (age, gender, smoking pack years, and Δbody weight) together with disease-related variables (mSASSS at baseline and one of the following: baseline BASDAI or ASDAS-CRP, mean CRP or mean ESR during follow-up, or ΔCRP or ΔESR) as well as medications (NSAID, bisphosphonates, and TNFi) that were hypothesized to influence changes in BMD. Mean CRP/ΔCRP or mean ESR/ΔESR was chosen depending on which gave the best model. Baseline BMD at the same measuring site and time between DXA measurements were also included in the models. Sex and menopause correlated too closely with each other to be included in the same model, and thus were used in separate models. There was no multicollinearity and residuals were analyzed. All tests were two-tailed and *p* ≤ 0.05 was considered statistically significant. Bonferroni correction was used for multiple comparisons.

## Results

### Patients

**Fig. 1:**
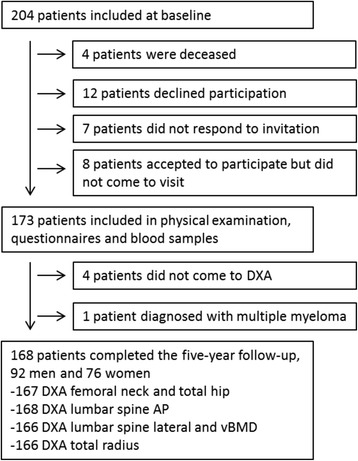
Flowchart of participation from baseline to the 5-year follow-up. *AP* anterior-posterior, *DXA* dual energy x-ray absorptiometry, *vBMD* volumetric bone mineral density

Of the 204 patients from baseline, 168 (82%) patients (55% men) completed all examinations at the 5-year follow-up (Fig. 1). Two patients had to be excluded from the lateral examinations due to insufficient quality, but the rest of the patients had three vertebrae legible in this projection.

Baseline and follow-up characteristics as well as medications are reported in Table 1. The mean age did not differ between men and women (49 ± 13 years vs 51 ± 13 years, *p* = 0.50), and neither did the duration of symptoms (24 ± 13 years for both sexes, *p* = 0.73).

**Table 1:** Characteristics of 168 patients with ankylosing spondylitis at baseline and at 5-year follow-up

	Baseline	5-year follow-up	*p* value
Demographic variables			
Sex, male/female	92 (55)/76 (45)		
Age, years	50 ± 13	55 ± 13	<0.001
Postmenopausal women	38/76 (50)	47/76 (62)	0.004
Current smokers	17 (10)	13 (8)	0.4
Pack years	5.5 ± 10.0	7.0 ± 11.3	< 0.001
Weight, kg	77 ± 16	79 ± 17	< 0.001
Height, cm	172 ± 10	171 ± 10	< 0.001
Time between DXA measurements, months		60.0 ± 2.0	
Disease-related variables			
Duration of symptoms, years	24 ± 13	29 ± 13	< 0.001
History of anterior uveitis	87 (52)	97 (58)	0.002
History of peripheral arthritis	98 (58)	106 (63)	0.008
History of coxitis	13 (8)	17 (10)	0.1
BASMI, score	3.1 ± 1.6	3.5 ± 1.6	< 0.001
BASFI, score	2.5 ± 2.0	2.7 ± 2.1	0.1
BASDAI, score	3.4 ± 2.1	3.5 ± 2.0	0.4
ASDAS-CRP, score	2.1 ± 0.9	2.1 ± 0.9	0.8
CRP, mg/L	5.5 ± 8.4	4.7 ± 5.2	0.2
Mean CRP last 5 years, mg/L		5.8 ± 5.9	
ESR, mm/h	14.2 ± 11.2	11.2 ± 10.4	0.001
Mean ESR last 5 years, mm/h		12.4 ± 8.7	
mSASSS, score	15.0 ± 20.0	16.6 ± 20.9	< 0.001
HLA-B27 positive	145 (86)		
Medications			
Patients on NSAIDs at visit	131 (78)	112 (67)	0.004
Exposure to NSAIDs during follow-up		145 (86)	
NSAID-index during follow-up, 0–100		34.5 ± 37.1	
Patients on TNFi at visit	33 (20)	38 (23)	0.3
Exposure to TNFi during follow-up		49 (29)	
Use of TNFi during follow-up, 0–1		0.2 ± 0.4	
Patients on GC at visit	5 (3)	3 (2)	0.6
Exposure to GC during follow-up		30 (18)	
Patients on bisphosphonate at visit	7 (4)	8 (5)	1.00
Exposure to bisphosphonate during follow-up		30 (18)	
Use of bisphosphonate during follow-up, 0–1		0.1 ± 0.3	
Patients on MHT at visit	6/76 (8)	4/76 (5)	0.7
Exposure to MHT during follow-up		9/76 (12)	

Values are mean ± SD or numbers of patients (%)

*ASDAS-CRP* Ankylosing Spondylitis Disease Activity Score based on C-reactive protein, *BASDAI* Bath Ankylosing Spondylitis Disease Activity Index, *BASFI* Bath Ankylosing Spondylitis Functional Index, *BASMI* Bath Ankylosing Spondylitis Metrology Index, *CRP* C-reactive protein, *DXA* dual-energy x-ray absorptiometry, *ESR* erythrocyte sedimentation rate, *GC* glucocorticoid, *MHT* menopausal hormone therapy, *mSASSS* modified Stoke Ankylosing Spondylitis Spine Score, *NSAID* nonsteroidal anti-inflammatory drug, *TNFi* tumor necrosis factor inhibitor

### Five-year BMD changes

**Fig. 2:**
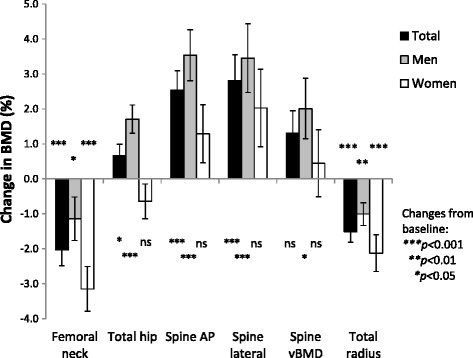
Mean change (percent) in bone mineral density over 5 years in AS patients, for the total group and divided by sex at the different measuring sites. Error bars represent SEM. *AP* anterior-posterior, *BMD* bone mineral density, *ns* not significant, *vBMD* volumetric BMD

Over 5 years, significant changes in BMD occurred at all five different measuring sites for the total group. For both sexes, BMD decreased at the femoral neck and the total radius. At the total hip and for the AP and lateral projections (including vBMD) of the spine BMD increased, changes that were statistically significant only in men (Fig. 2). The Pearson correlation coefficient for BMD at AP and lateral spine was 0.68 at both baseline and follow-up and 0.84 for ΔBMD (*p* < 0.001).

### Prevalence of low BMD

At baseline, 23% of the patients had osteoporosis according to the WHO definition or BMD below the expected range for age at any measuring site compared to 27% at follow-up, while 35% had osteopenia or Z-score < –1 SD at baseline compared to 32% at follow-up. These prevalences had not changed significantly (*p* = 0.34 for osteopenia) from baseline. Women had higher prevalence of osteoporosis/BMD below the expected range for age than men, both at baseline and follow-up (Table 2).

**Table 2:** Frequency of osteoporosis at baseline and at the 5-year follow-up

Measuring site	Group	Patients with osteoporosis/BMD below expected range for age		*p* value
		Baseline	5-year follow-up	
Any measuring site	Total	39 (23)	45 (27)	0.24
	Men	12 (13)**	16 (17)**	0.29
	Women	27 (36)	29 (38)	0.75
Femoral neck	Total	10 (6)	12 (7)	0.72
	Men	6 (7)	7 (8)	1.00
	Women	4 (5)	5 (7)	1.00
Total hip	Total	1 (1)	1 (1)	1.00
	Men	1 (1)	1 (1)	1.00
	Women	0 (0)	0 (0)	–
Spine AP	Total	16 (10)	12 (7)	0.39
	Men	4 (4)*	2 (2)**	0.69
	Women	12 (16)	10 (13)	0.69
Spine lateral	Total	NA	NA	NA
	Men	NA	NA	NA
	Women	20 (26)	18 (24)	0.63
Total radius	Total	13 (8)	20 (12)	**0.04**
	Men	7 (8)	11 (12)	0.13
	Women	6 (8)	9 (12)	0.38

Values are numbers (%)

*p < 0.05, **p < 0.01, versus women

Significant *p* values are shown in bold typeface

*AP* anterior-posterior, *BMD* bone mineral density, *NA* not available

The total AS group did not differ significantly from the reference group at any measuring site for BMD at baseline. At the 5-year follow-up, the total group had significantly higher BMD than the reference group at the total hip and AP lumbar spine (*p* = 0.002 and 0.001, respectively) (Table 3).

**Table 3:** Bone mineral density, T-score and Z-score at baseline and the 5-year follow-up

Site	Group	Baseline BMD, g/cm^2^	Follow-up BMD, g/cm^2^	Baseline T-score, SD	Follow-up T-score, SD	Baseline Z-score, SD	Follow-up Z-score, SD
Femoral neck	Total	0.78 ± 0.13	0.77 ± 0.13***	–0.9 ± 1.0	–1.0 ± 1.0***	–0.1 ± 1.0	–0.1 ± 0.9
	Men	0.80 ± 0.13	0.79 ± 0.12*	–0.9 ± 0.9	–1.0 ± 0.9	–0.2 ± 0.9	–0.2 ± 0.9
	Women	0.76 ± 0.13	0.73 ± 0.13***	–0.8 ± 1.1	–1.0 ± 1.1***	0.0 ± 1.0	0.0 ± 1.0
Total hip	Total	0.94 ± 0.14	0.94 ± 0.14*	–0.4 ± 0.9	–0.4 ± 1.0	0.0 ± 0.9	0.2 ± 0.9***
	Men	0.98 ± 0.13	0.99 ± 0.13***	–0.4 ± 0.9	–0.3 ± 0.9***	–0.1 ± 0.9	0.2 ± 0.9***
	Women	0.89 ± 0.13	0.88 ± 0.13	–0.4 ± 1.0	–0.5 ± 1.1	0.1 ± 0.9	0.3 ± 1.0***
Lumbar spine AP	Total	1.03 ± 0.18	1.05 ± 0.19***	–0.4 ± 1.5	–0.2 ± 1.6***	0.2 ± 1.5	0.6 ± 1.6***
	Men	1.08 ± 0.17	1.11 ± 0.18***	–0.1 ± 1.6	0.2 ± 1.6***	0.2 ± 1.7	0.7 ± 1.7***
	Women	0.96 ± 0.16	0.97 ± 0.17	–0.8 ± 1.4	–0.7 ± 1.5	0.1 ± 1.4	0.5 ± 1.5***
Lumbar spine lateral	Total	0.72 ± 0.13	0.74 ± 0.14***	NA	NA	NA	NA
	Men	0.76 ± 0.12	0.79 ± 0.14***	NA	NA	NA	NA
	Women	0.68 ± 0.13	0.69 ± 0.13	–1.7 ± 1.5	–1.6 ± 1.6	–0.1 ± 1.4	0.4 ± 1.5***
Lumbar spine vBMD	Total	0.19 ± 0.03	0.19 ± 0.03	NA	NA	NA	NA
	Men	0.19 ± 0.03	0.19 ± 0.03*	NA	NA	NA	NA
	Women	0.19 ± 0.04	0.20 ± 0.04	NA	NA	NA	NA
Total radius	Total	0.60 ± 0.08	0.59 ± 0.08***	–0.7 ± 1.1	–0.8 ± 1.2***	–0.0 ± 1.0	0.0 ± 1.1
	Men	0.65 ± 0.06	0.64 ± 0.06**	–0.7 ± 1.1	–0.8 ± 1.1**	–0.2 ± 1.0	–0.2 ± 1.1
	Women	0.54 ± 0.06	0.53 ± 0.07***	–0.6 ± 1.1	–0.8 ± 1.3***	0.2 ± 0.9	0.3 ± 1.0

Values are mean ± SD

*p < 0.05, **p < 0.01, ***p < 0.001, versus baseline

*AP* anterior-posterior, *BMD* bone mineral density, *NA* not available, *SD* standard deviation, *vBMD* volumetric BMD

### Predictors for changes in BMD

The results from the multiple linear regression analyses with ΔBMD at the various measuring sites as a dependent variable are shown in Table 4. Higher mean CRP during follow-up was associated with decreasing femoral neck BMD. Decreases in ESR were associated with increases in BMD at the total hip, and the AP, lateral, and vBMD lumbar spine (similar results were seen for ΔCRP but resulted in lower *R* ^2^ values). No associations with ΔBMD were observed for mean ESR, baseline BASDAI, or ASDAS-CRP. Low mSASSS was a predictor for BMD decrease in the femoral neck. On exchanging mSASSS to lumbar mSASSS as a covariate, no association with ΔBMD was seen. Increased weight was associated with increased BMD at all measuring sites except the femoral neck and radius.

**Table 4:** Multiple linear regression analyses with ΔBMD (change in %) as a dependent variable

	Femoral neck		Total hip		Spine AP		Spine lateral		Spine vBMD		Total radius	
Constant	20.4		1.5		–13.4		–47.3		–40.5		10.4	
	β	*p*	β	*p*	β	*p*	β	*p*	β	*p*	β	*p*
Baseline												
BMD at same site, g/cm^2^	**–8.24**	**0.036**	0.98	0.67	0.80	0.80	0.81	0.89	–16.3	0.42	2.89	0.60
Age, years	**–0.12**	**0.006**	–0.05	0.063	–0.04	0.31	–0.04	0.50	–0.07	0.22	**–0.11**	**< 0.001**
Sex	–1.61	0.11	**–1.68**	**0.007**	–2.06	0.051	–1.70	0.27	–1.47	0.25	–0.47	0.58
Pack years	**0.13**	**0.004**	**0.08**	**0.003**	0.09	0.054	0.12	0.086	0.09	0.12	–0.04	0.16
mSASSS, score	**0.06**	**0.041**	0.005	0.76	–0.03	0.27	–0.08	0.052	–0.06	0.10	0.02	0.26
During follow-up												
Time between DXA, months	–0.19	0.41	–0.02	0.86	0.24	0.31	**0.81**	**0.016**	**0.77**	**0.009**	–0.14	0.36
Δweight, kg	0.03	0.72	**0.24**	**< 0.001**	**0.37**	**< 0.001**	**0.49**	**< 0.001**	**0.51**	**< 0.001**	0.08	0.18
NSAID-index, 0–100	0.02	0.064	0.01	0.097	0.01	0.42	0.01	0.56	0.00	0.89	0.01	0.26
Use of TNFi, 0–1	–1.53	0.11	1.23	0.086	**3.15**	**0.012**	2.17	0.22	1.06	0.49	–0.65	0.41
Use of bisphosphonate, 0–1	**4.64**	**0.013**	**5.61**	**< 0.001**	**13.7**	**< 0.001**	**15.5**	**< 0.001**	**11.6**	**< 0.001**	0.83	0.48
Mean CRP last 5 years, mg/L	**–0.15**	**0.046**	NA		NA		NA		NA		–0.09	0.072
ΔESR, mm/h	NA		**–0.10**	**< 0.001**	**–0.12**	**0.004**	**–0.16**	**0.007**	**–0.13**	**0.012**	NA	
*R* ^2^	0.22		0.44		0.41		0.33		0.34		0.20	

β are unstandardized coefficients

Coding for sex: man = 0, woman = 1

Significant β and *p* values are shown in bold typeface

*AP* anterior-posterior, *BMD* bone mineral density, *CRP* C-reactive protein, *DXA* dual-energy x-ray absorptiometry, *ESR* erythrocyte sedimentation rate, *mSASSS* modified Stoke Ankylosing Spondylitis Spine Score, *NA* not available, *NSAID* nonsteroidal anti-inflammatory drug, *TNFi* tumor necrosis factor inhibitor, *vBMD* volumetric BMD

Male sex predicted an increase in total hip BMD. If sex was changed to menopause as a covariate, significance was only seen for menopause at the total radius (β = –2.04, *p* = 0.036). Menopause during follow-up was not a significant covariate. A multivariate regression model including synovitis and uveitis was performed; they were not significant covariates and did not improve prediction of the model.

Exposure to medication was related to ΔBMD; use of bisphosphonate predicted an increase in BMD at all measuring sites except the radius. Use of TNFi predicted an increase in AP lumbar spine BMD (Table 4).

To further study if osteoproliferation had an impact on ΔBMD, patients < 50 years with syndesmophytes at baseline were compared with patients without syndesmophytes. The osteoproliferative group had higher ΔBMD in the AP spine and total hip (Table 5). However, in regression analyses, no association with mSASSS and ΔBMD was seen. In the group with syndesmophytes 52% of the patients had exposure of TNFi during follow-up compared to 26% in those without syndesmophytes (*p* = 0.036). The exposure of bisphosphonates also differed between the groups; 19% of patients with syndesmophytes vs 3.3% without syndesmophytes (*p* = 0.026).

**Table 5:** Comparing percent change in BMD in patients < 50 years without and with baseline syndesmophytes

Measuring site	% change in BMD		*p* value between groups
	No syndesmophytes (*n* = 59)	Syndesmophytes present (*n* = 27)	
Spine AP	1.13 ± 5.4*	6.44 ± 9.6**	**0.01**
Spine lateral	2.12 ± 8.0	6.04 ± 11.5*	0.19
vBMD	1.08 ± 7.6	4.15 ± 10.0	0.31
Total hip	–0.02 ± 3.6	2.84 ± 3.7**	**0.001**
Femoral neck	–3.15 ± 5.2***	–0.48 ± 5.0	**0.04**
Total radius	–0.35 ± 2.9	–0.68 ± 3.1	0.80

Values are presented as mean ± SD

*p < 0.05, **p < 0.01, ***p < 0.001, versus baseline

Significant *p* values are shown in bold typeface

*AP* anterior-posterior, *BMD* bone mineral density, *vBMD* volumetric BMD

### Subgroup analyses of different medications

**Fig. 3:**
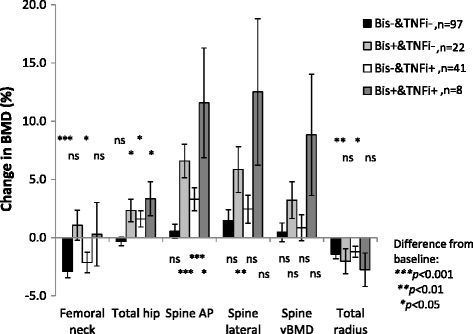
Mean change (percent) in bone mineral density over 5 years according to ever exposure to medication during follow-up; patients not exposed to bisphosphonates or TNFi, patients ever exposed to bisphosphonates but not to TNFi during follow-up, patients ever exposed to TNFi but not bisphosphonates during follow up, and patients ever exposed to both TNFi and bisphosphonate during follow-up. Error bars represent SEM. *AP* anterior-posterior, *Bis* bisphosphonate, *BMD* bone mineral density, *ns* not significant, *TNFi* tumor necrosis factor inhibitor, *vBMD* volumetric BMD

To further study the impact of treatment on ΔBMD, subgroup analyses for different treatment groups were performed in the total group of patients. Patients exposed neither to bisphosphonates nor TNFi during follow-up were compared with patients exposed to bisphosphonates, to patients exposed to TNFi, and to patients exposed to both bisphosphonates and TNFi (Fig. 3).

BMD decreased significantly at the femoral neck and the total radius in patients without bisphosphonates but was stable in patients exposed to bisphosphonates. Compared to patients exposed to bisphosphonates, patients without exposure to bisphosphonates and TNFi had significantly decreased femoral neck BMD (difference in ΔBMD 4%, *p* = 0.03) (Fig. 3).

All patients with exposure to bisphosphonates and/or TNFi had significantly increased AP lumbar spine and total hip BMD compared with non-exposed patients (*p* < 0.001 to 0.048) except at the total hip for combination therapy (*p* = 0.075). There was no significant difference for ΔBMD at the lateral spine between non-exposed patients and patients exposed to bisphosphonates and/or TNFi (Fig. 3).

The group without exposure to medication was further studied with the same regression models (except treatments) as described previously. Higher mean ESR during follow-up was associated with a decrease in BMD at all sites except for the AP spine and total radius (Table 6).

**Table 6:** Multiple linear regression analyses, ΔBMD (%) as adependent variable, with no exposure to TNFi or bisphosphonates.

Constant	Femoral neck		Total hip		Spine AP		Spine lateral		Spine vBMD		Total radius	
	14.0		5.53		–16.4		–54.4		–47.6		3.58	
	β	*p*	β	*p*	β	*p*	β	*p*	β	*p*	β	*p*
Baseline												
BMD at same site, g/cm^2^	0.09	1.0	4.90	0.1	3.20	0.4	–6.94	0.3	–47.9	0.06	9.73	0.2
Age, years	–0.08	0.1	–0.02	0.6	0.03	0.6	0.03	0.7	–0.05	0.5	**–0.11**	**0.005**
Sex	–0.53	0.7	–0.75	0.4	–2.11	0.1	–1.42	0.5	–0.40	0.8	0.15	0.9
Pack years	**0.17**	**0.01**	0.07	0.08	0.12	0.07	**0.20**	**0.04**	0.16	0.07	**–0.09**	**0.03**
mSASSS, score	0.08	0.06	0.01	0.7	–0.07	0.1	–0.07	0.3	–0.03	0.6	0.02	0.5
During follow-up												
Time between DXA, months	–0.27	0.4	–0.16	0.4	0.21	0.5	**1.00**	**0.02**	**1.00**	**0.006**	–0.09	0.6
Δweight, kg	0.05	0.7	**0.25**	**0.001**	**0.38**	**0.001**	**0.60**	**0.001**	**0.61**	**0.001**	0.04	0.5
NSAID-index, 0–100	0.01	0.3	0.01	0.4	0.03	0.08	0.03	0.2	0.021	0.3	0.01	0.2
Mean ESR last 5 years, mm/h	**–0.19**	**0.01**	**–0.11**	**0.02**	–0.09	0.3	**–0.24**	**0.04**	**–0.21**	**0.04**	NA	
Mean CRP last 5 years, mg/L	NA		NA		NA		NA		NA		–0.14	0.06
*R* ^2^	0.21		0.32		0.27		0.28		0.34		0.29	

Betas are unstandardized coefficients

Coding for sex: 0 = man, 1 = woman

Number of patients = 97

Significant *p* values are shown in bold typeface

*AP* anterior-posterior, *BMD* bone mineral density, *CRP* C-reactive protein, *DXA* dual-energy x-ray absorptiometry, *ESR* erythrocyte sedimentation rate, *mSASSS* modified Stoke Ankylosing Spondylitis Spine Score, *NA* not available, *NSAID* nonsteroidal anti-inflammatory drug, *TNFi* tumor necrosis factor inhibitor, *vBMD* volumetric BMD

### Vertebral fractures

Of 17 (10%) patients with VF at baseline, only three (1.8%) had progressed in Genant score. Two patients without fractures at baseline had developed vertebral fractures at follow-up, both in the lumbar spine. One of them developed a grade 1 VF and the other patient developed two grade 2 fractures. The third patient already had four VF at baseline located in the thoracic spine and developed one new VF grade 2 in the thoracic spine and worsened from grade 2 to grade 3 in one existing VF. Excluding patients with VF did not alter the ΔBMD at the different measuring sites for the group. There were too few patients with new VF to assess VF predictors.

## Discussion

The current study has shown that BMD in AS patients after 5 years decreased at the femoral neck and total radius and increased at the total hip and the lumbar spine, both for AP and lateral projections. Inflammation as measured by CRP or ESR was associated with a decrease in BMD. Previous longitudinal BMD studies in AS patients have mainly assessed BMD at the hip region and the AP projection of the lumbar spine [9–11, 27]. One study of early inflammatory back pain included the hand [13] and another included the forearm [12]. One previous study, in line with our findings, reported reduction of femoral neck BMD and a more pronounced decrease in BMD in patients with high ESR during follow-up [11]. Two studies only have reported a significant reduction of femoral neck BMD in patients with active disease during follow-up [9, 13]. Wang et al. reported BMD increases in both the femoral neck and forearm; however, in regression analyses high ESR at baseline was associated with decreased BMD in the femur [12]. Haugeberg et al. found no change in ΔBMD of the hands [13]. The small decrease in BMD at the total radius in the current study should be interpreted with caution considering the relatively large CV at this site. The only predictors for a decrease in BMD at the total radius in the current study were age and menopause.

In the current study, in contrast to decreasing femoral neck BMD, total hip BMD increased, especially for younger patients with syndesmophytes who also had a greater exposure to TNFi and bisphosphonates compared to those without syndesmophytes. The total hip has been studied less longitudinally than the femoral neck in AS patients. Previous studies have shown decreased BMD [10, 13]. Our patients also had higher total hip BMD at follow-up compared with the reference group. Increased BMD was seen in patients exposed to bisphosphonates and/or TNFi. The part of the total hip that showed increased BMD was the trochanter. Trochanteric BMD has been shown to increase more with bisphosphonates than femoral neck BMD [28]. In the current study, the hip was not radiographed, and thus arthritic changes cannot be accounted for. The results in the current study suggest that the femoral neck is the best site for assessing bone loss in AS patients.

In the spine, BMD in both the AP and lateral projections increased during follow-up. The increase in the AP projection may be due to the osteoproliferation that may occur in the spine in AS patients [1, 7]. Use of the lateral projection of the lumbar spine has been suggested to exclude much of the osteoproliferative changes seen in AS. Two cross-sectional studies have shown lower lumbar BMD in the lateral projection but not in the AP projection when comparing AS patients with healthy controls [29, 30]. A report on baseline data in the current study showed that AP lumbar spine BMD was significantly higher than lateral spine BMD [31]. Our current study is the first to evaluate the lateral projection longitudinally in AS patients and it was unexpected to find increasing BMD. Since we have no reference values for lateral BMD measurements for men, and since there is sparse knowledge in the literature how spinal lateral BMD changes over time in the general population, our results have to be interpreted with caution. Previous studies in the general population have shown increasing AP BMD but decreasing lateral BMD over time in older men [14, 15], whereas postmenopausal women showed an increase in both AP and lateral BMD [15]. Perimenopausal women showed a decrease in AP BMD but had a statistically nonsignificant decrease in lateral BMD [32]. Nevertheless, the increase in AP and lateral BMD in this current study was significant for men. Men also had significantly higher mSASSS score than women. We were not able to show an association between osteoproliferation and increased spinal BMD, except in younger patients in the AP projection. In a study by Kaya et al., AS patients had increased AP spine BMD and SASSS over 2 years, without a correlation between the changes [10]. Wang et al. reported increased AP spine BMD, but performed no analysis to evaluate an association between osteoproliferation and ΔBMD [12]. Tan et al. recently reported improved detection of syndesmophytes by computed tomography compared to x-ray and further reported that syndesmophytes may form around the intervertebral discs [33, 34]. Thus, osteoproliferation is underestimated by mSASSS and the lateral projection of the lumbar spine DXA does not exclude all osteoproliferation. Using dual-energy quantitative computed tomography (DEQCT), Karberg et al. suggested that bone growth and bone loss occur in parallel in the spine in AS patients [35]. We suggest that it would be of great value to examine AS patients longitudinally with both DXA and QCT in order to better separate vertebral cortical and trabecular bone loss and bone proliferation. Treatment was also shown to have an influence on the increases in spinal BMD. Use of TNFi was a predictor for increases in AP BMD, and bisphosphonates predicted increases in both AP and lateral spine BMD. Patients without such treatments did not show an increase in the spinal BMD. However, our study was not designed to study treatment effects, and the use of propensity score matching was not considered relevant due to low number of TNFi-exposed patients who also had a substantial variation in treatment duration and different starting points.

In the multiple linear regression analyses we found increased weight during follow-up to be associated with increased BMD at the total hip and spine. Both lean and fat mass are known determinants of bone mass and have a positive effect on BMD [36]. On the other hand, fat can also cause bias in the measurements and overestimate BMD [37]. Irrespective of mechanism, the estimates for the other predictors are likely to be valid since change in weight was adjusted for in the multiple linear regression analyses.

Exposure to bisphosphonates was shown to have a positive impact on BMD in the spine, the total hip and, to a certain extent, at the femoral neck. In a retrospective study on AS patients grouped according to treatment agents, no differences between the treatment groups were found for ΔBMD except for increased trochanteric BMD if the patients had received both bisphosphonates and TNFi [27]. A randomized placebo-controlled trial on the effect of alendronic acid on BMD in non-osteoporotic AS patients found no difference in ΔBMD in the two groups after 1 year [38]. TNFi therapy has been shown to improve lumbar spine and total hip BMD in some longitudinal studies in line with our findings [39]. Concerning NSAIDs, we found no significant effect on BMD related to NSAIDs. Results on the effect of NSAIDs on BMD in the general population in observational studies are conflicting [40–42]. The subject is sparsely studied in spondyloarthritis. One recent longitudinal study reported a protective effect of NSAID use on hip BMD in patients with early inflammatory back pain [43], but the result needs to be confirmed.

The clinical concern with low BMD is the risk for fractures. Previous studies on patients with AS have shown associations with low BMD at the spine and hip region and with prevalent VF [44–46]. In one of these studies new or worsening of VF was associated with both lower lumbar spine and hip BMD at baseline [45]. The association of BMD loss over time and development of VF has not been investigated in AS patients, and in this current study it is not possible to elucidate the clinical implications of the bone loss observed at the femoral neck.

One limitation with our study is the lack of a control group for BMD measurements. However, the current study, with a wide age range, would have needed a large control group. The large age- and sex-matched reference material in the DXA scanner was deemed sufficient. Also, using Z-scores, results from other studies can be compared to the current results. Another limitation is the lack of a reference material for the lateral projection for men.

Strengths of this study are the long follow-up time and the large and well characterized patient group with many factors, potentially able to affect BMD, identified and analyzed.

## Conclusion

After 5 years BMD decreased at the femoral neck and the total radius and increased at the lumbar spine in AS patients, both in the AP and the lateral projections. The increases in the lateral projection, as well as in the AP projection, might be influenced by osteoproliferation and we suggest that the best site to assess bone loss in AS patients is the femoral neck. This study also indicates that inflammation has an adverse effect and that bisphosphonates and TNFi have a positive impact on BMD in AS patients.

## Abbreviations

AP: Anterior-posterior
AS: Ankylosing spondylitis
ASAS: Assessment of SpondyloArthritis International Society
ASDAS-CRP: Ankylosing Spondylitis Disease Activity Score based on C-reactive protein
BASDAI: Bath Ankylosing Spondylitis Disease Activity Index
BASFI: Bath Ankylosing Spondylitis Functional Index
BAS-G: Bath Ankylosing Spondylitis Patient Global Score
BASMI: Bath Ankylosing Spondylitis Metrology Index
BMD: Bone mineral density
CRP: C-reactive protein
CV: Coefficient of variation
DEQCT: Dual-energy quantitative computed tomography
DXA: Dual-energy x-ray absorptiometry
ESR: Erythrocyte sedimentation rate
mSASSS: Modified Stoke Ankylosing Spondylitis Spinal Score
NSAID: Nonsteroidal anti-inflammatory drug
SASSS: Stoke Ankylosing Spondylitis Spine Score
SD: Standard deviation
SEM: Standard error of the mean
TNFi: Tumor necrosis factor inhibitors
vBMD: Volumetric bone mineral density
VF: Vertebral fracture
WHO: World Health Organization
